# 452. Ongoing Surveillance of SARS-CoV-2 for *In Silico* Analysis to Monitor the Performance of SARS-CoV-2 Assays in BIOFIRE® Panels

**DOI:** 10.1093/ofid/ofad500.522

**Published:** 2023-11-27

**Authors:** Eleanor K Horrocks, Toma Todorov, Alexandra Debernardi, Usha Spaulding, Tanner Robinson, Jeremiah Antosch, Zhenmei Lu, Matthew Jones, Joann Cloud

**Affiliations:** bioMérieux, Salt Lake City, Utah; bioMérieux, Salt Lake City, Utah; bioMérieux, Salt Lake City, Utah; bioMérieux, Salt Lake City, Utah; bioMérieux, Salt Lake City, Utah; bioMérieux, Salt Lake City, Utah; bioMérieux, Salt Lake City, Utah; bioMérieux, Salt Lake City, Utah; bioMérieux, Salt Lake City, Utah

## Abstract

**Background:**

In response to the COVID-19 pandemic, bioMérieux has incorporated assays to detect SARS-CoV-2 into the BIOFIRE® Respiratory 2.1 (RP2.1) Panel, the BIOFIRE® SPOTFIRE® Respiratory (R) Panel and the BIOFIRE® SPOTFIRE® Respiratory (R) Panel Mini. All panels use two assays for SARS-CoV-2 detection, each targeting a different gene. Positive detection in only one of the two assays is required for a positive SARS-CoV-2 result. As new variants of SARS-CoV-2 emerge, *in silico* analysis remains crucial to ensure that both SARS-CoV-2 assays are reactive to circulating strains.

**Methods:**

Sequence data (https://gisaid.org/) for SARS-CoV-2 variants deemed significant by the WHO and UKHSA are analyzed monthly. Assay primer regions are assessed for mismatches using Geneious Prime® as well as proprietary software tools. When primer mismatches with potential to affect the sensitivity of SARS-CoV-2 detection are identified (mismatches occurring in the 3’ half of a primer in both assays), and meet the testing criteria, the BIOFIRE RP2.1 Panel is used to empirically evaluate the impact by comparing synthetic templates with mismatches of concern to synthetic template without mismatches.

**Results:**

As of March 21, 2023, nearly 1,000 variants and 12,744,136 sequences have been analyzed. Of these, only 481 sequences (0.0038%) contain paired mismatches of concern. Wet testing showed < 10x lower projected sensitivity for 288 (72 unique) sequences, 10-100x lower sensitivity for 16 (10 unique) sequences, and only 1 sequence had 100-1000x lower projected sensitivity. Lineage defining mutations were seen within a SARS-CoV-2 assay primer region in 27 variants. However, most lack mutations in the second assay, indicating no major risk for SARS-CoV-2 detection. Wet testing shows that the inclusion of two SARS-CoV-2 assays in the panels helps to mitigate the effect of mismatches of concern.

**Conclusion:**

Based on the comprehensive *in silico* analysis of available sequences and variants, BIOFIRE RP2.1 Panel, SPOTFIRE R Panel and SPOTFIRE R Panel Mini continue to function as intended with >99.99% detection of SARS-CoV-2 sequences.

**Disclosures:**

**Eleanor K. Horrocks**, bioMerieux: employee|bioMerieux: Stocks/Bonds **Toma Todorov, n/a**, bioMerieux: employee|bioMerieux: Stocks/Bonds **Alexandra Debernardi, n/a**, bioMerieux: employee|bioMerieux: Stocks/Bonds **Usha Spaulding, n/a**, bioMerieux: employee|bioMerieux: Stocks/Bonds **Tanner Robinson, n/a**, bioMerieux: employee|bioMerieux: Stocks/Bonds **Jeremiah Antosch, n/a**, bioMerieux: employee|bioMerieux: Stocks/Bonds **Zhenmei Lu, n/a**, bioMerieux: employee|bioMerieux: Stocks/Bonds **Matthew Jones, MS**, bioMerieux: employee|bioMerieux: Stocks/Bonds **Joann Cloud, PhD**, bioMerieux: employee|bioMerieux: Stocks/Bonds

